# Microbial Associations with Pancreatic Cancer: A New Frontier in Biomarkers

**DOI:** 10.3390/cancers13153784

**Published:** 2021-07-27

**Authors:** Mark Stasiewicz, Marek Kwaśniewski, Tomasz M. Karpiński

**Affiliations:** 1 Research Group of Medical Microbiology, Chair and Department of Medical Microbiology, Poznań University of Medical Sciences, Wieniawskiego 3, 61-712 Poznań, Poland; 84682@student.ump.edu.pl; 2 Chair and Department of Medical Microbiology, Poznań University of Medical Sciences, Wieniawskiego 3, 61-712 Poznań, Poland; mkwasniewski@ump.edu.pl

**Keywords:** pancreatic cancer, pancreas, cancer, cancer screening, microbiota, dysbiosis, *Porphyromonas*, oral cavity, *Helicobacter*

## Abstract

**Simple Summary:**

Pancreatic cancer (PC) continues to be characterized by high morbidity and mortality, owing to the fact, among others, that it is often diagnosed at late stages. Thus far, the search for reliable biomarkers has failed. A number of recent studies have found that there are differences in the microbiota between patients with PC and their healthy counterparts. These differences extend to specific anatomical locations such as the oral cavity, the gastrointestinal tract, and the pancreas itself. The purpose of this review is to outline some of the main differences in the bacterial and fungal populations between patients with PC and their healthy counterparts that have recently come to light. Additionally, the present review aims to highlight the mechanisms underlying the aforementioned microbial associations with PC.

**Abstract:**

Pancreatic cancer (PC) remains a global health concern with high mortality and is expected to increase as a proportion of overall cancer cases in the coming years. Most patients are diagnosed at a late stage of disease progression, which contributes to the extremely low 5-year survival rates. Presently, screening for PC remains costly and time consuming, precluding the use of widespread testing. Biomarkers have been explored as an option by which to ameliorate this situation. The authors conducted a search of available literature on PubMed to present the current state of understanding as it pertains to the use of microbial biomarkers and their associations with PC. Carriage of certain bacteria in the oral cavity (e.g., *Porphyromonas gingivalis*, *Aggregatibacter actinomycetemcomitans*, *Streptococcus* sp.), gut (e.g., *Helicobacter pylori*, Synergistetes, Proteobacteria), and pancreas (e.g., *Fusobacterium* sp., Enterobacteriaceae, Pseudomonadaceae) has been associated with an increased risk of developing PC. Additionally, the fungal genus *Malassezia* has likewise been associated with PC development. This review further outlines potential oncogenic mechanisms involved in the microbial-associated development of PC.

## 1. Introduction

Pancreatic cancer (PC) remains a major health concern across much of the globe. As of 2020, it was the 14th most common cancer (with an estimated 495,733 new cases and 466,003 new deaths reported in 2020) [1]. However, it is expected to become the third most common cancer in the EU by 2025 [2]. The incidence and mortality of PC is rising in many countries; however, the highest rates are observed in high-income countries [3]. Males are disproportionately affected globally; however, the highest discrepancies are likewise observed in high-income regions with an age-standardized rate per 100,000 of 7.2 compared with 5.0 for females [1]. At present, the 5-year survival rate associated with PC is low, with most studies placing it at, or below, 10% in high-income countries [4,5]. Interestingly, the survival rates have not improved since the 1960s [6]. It is believed that this is due in large part to low early detection rates. Pancreatic adenocarcinoma (PDAC) is a malignant epithelial neoplasm, accounting for approximately 85% of all pancreatic tumors [7]. Consistent with pancreatic cancers in general, PDAC screening is lacking, with only about one-fifth of patients presenting with surgically resectable forms of the disease [8].

It is held that the reason underpinning the low survival rates is the fact that the majority of patients are initially diagnosed only after the neoplasia has become metastatic [9]. A study by Canto et al. revealed that 42% (92/216) of asymptomatic individuals at high risk for developing PC had at least one pancreatic mass or a dilated pancreatic duct [10]. These findings underscore the insidious nature of PC; however, they do not account for individuals with population risk of developing the disease. The late presentation and difficulty associated with diagnosing PC can be at least partially explained by the nonspecific symptoms and close association with major blood vessels [11]. The latter is believed to play an important role in the high metastatic nature of PC; this is further enhanced by lymph node metastasis and neural invasion [12,13]. Additionally, the relatively low incidence has led to recommendations against population-based screening with only certain at-risk groups having well-established screening protocols [14]. Presently, the main diagnostic modalities utilized to screen those suspected of having PC include: multi-detector computed tomography, magnetic resonance imaging, endoscopic ultrasound (+/− fine needle aspirate), and positron emission tomography [9]. All four of the above require specialized facilities and prove to be costly [15]. This situation could be at least partially alleviated by the identification of biomarkers for PC. Unfortunately, no reliable and cost-effective biomarkers have been identified to date.

For many years, the association between microorganisms and certain neoplastic diseases has been well established. Recently, the role of other microorganisms in the pathogenesis of various cancers has come under increased scrutiny. It has been estimated that between 7–20% of all cancers globally may be attributed to infectious agents [16]. The microbiome has been implicated in a number of cancers, many of which can be classified as gastrointestinal in nature. The most well-known example of this phenomenon is that of *Helicobacter pylori* and the direct role these bacteria play in the pathobiology of gastric cancer [17,18]. Additionally, a number of oral microorganisms have been found to contribute to cancer development. Several of these bacteria appear to be associated with neoplastic diseases such as oral squamous cell carcinoma, colorectal cancer, and PC [19]. Periodontitis and tooth loss have been found to be associated with an increased risk of developing PC, although there are issues with the way in which several of these studies adjusted for risk factors [20,21]. Furthermore, infectious agents from beyond the oral cavity have likewise been associated with the development and progression of PC [22].

There has been an increase in the number of studies examining microbial associations with PC in recent years. However, to the best of our knowledge, the present review is the first to summarize the data from across anatomical locations and provide possible mechanistic explanations for the observed associations. Thus, the aim of this review is to highlight the main microorganisms which are associated with an increased risk of developing PC. The present investigation will likewise highlight the main mechanisms by which the microorganisms in question are believed to drive the development and progression of PC. The PubMed database was used to search for articles using the keywords “microbiome”, “pancreas”, “pancreatic cancer”, “oral bacteria”, “oncogenic mechanisms”, “pancreatic cancer screening”, “periodontitis”, “gut”, and “*Helicobacter pylori*”, with more recent literature being favored. Additionally, a manual review of references from the literature obtained from PubMed searches was performed.

## 2. Microbial Associations with Pancreatic Cancer

The following section aims to highlight the associations between carriage of certain microbial populations in specific anatomical locations and the development of PC (Table 1). It must be noted that at present, available data are mostly limited to associations rather than causal relationships between the presence of specific microorganisms and the development of PC. Section 3 of the present review outlines possible mechanisms underlying some of the associations presented below.

### 2.1. Oral Cavity

The last several years have seen increased interest in the association between oral bacteria and PC. Several studies have found that the presence of *Porphyromonas gingivalis* in the oral cavity is associated with a higher incidence of PC [23,24]. Michaud et al. found that individuals with high levels of antibodies against *P*. *gingivalis* ATCC 53978 have a two-fold higher risk (odds ratio [OR] = 2.14) of developing PC than their counterparts with low levels of the same antibody [24]. In 2018, Fan et al., demonstrated that there is an increased risk of developing PC in patients with oral carriage of *Aggregatibacter actinomycetemcomitans* (OR = 2.20) and *Alloprevotella* (OR = 1.20) [23]. Furthermore, a recent prospective study by Wei et al. found that oral carriage of *Streptococcus* and *Leptotrichia* was associated with a higher risk of PDAC (OR = 5.344 and OR = 6.886, respectively) [25]. The same study demonstrated that *Veillonella* and *Neisseria* decreased the risk of PDAC (OR = 0.187 and OR = 0.309, respectively). The aforementioned relationships are further supported by an investigation from 2015 by Torres et al. which found that the *Leptotrichia* to *Porphyromonas* ratio was significantly higher in patients with PDAC [26].

**Figure 1:**
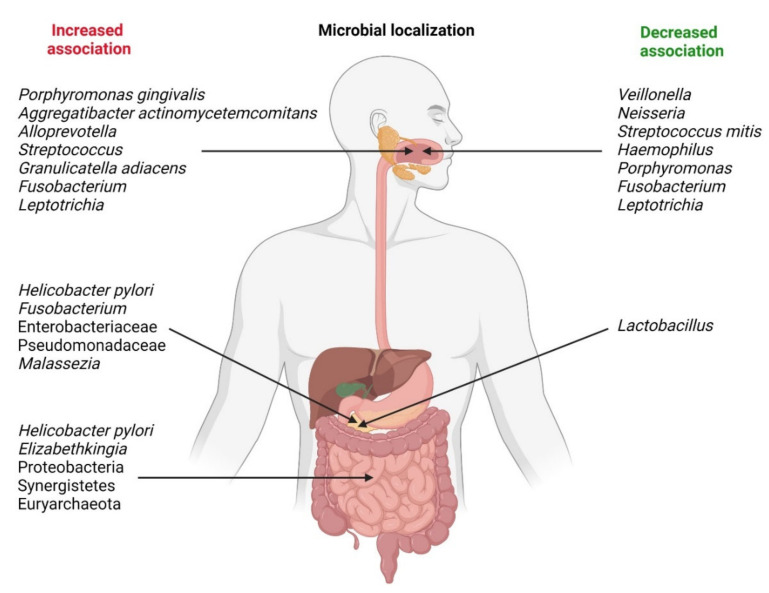
Microbial localization and changes in bacterial associations related with pancreatic carcinogenesis.

**Figure 2:**
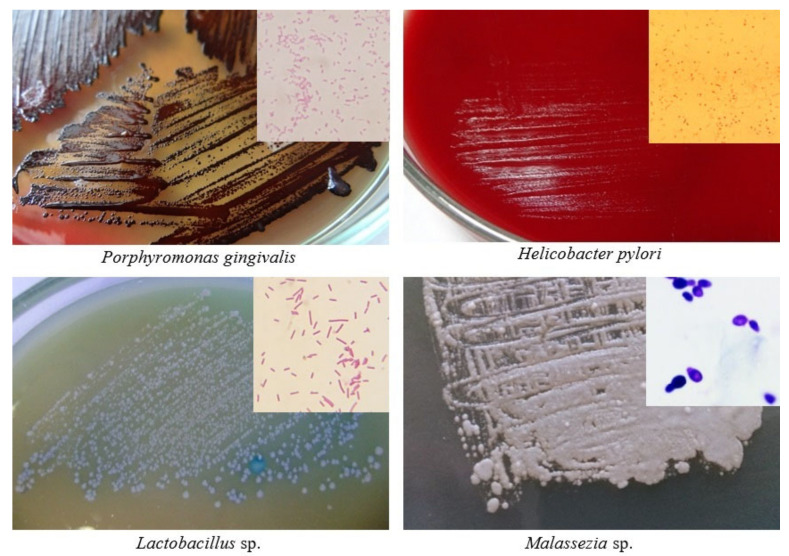
Cultures and Gram staining (in corners) of some microorganisms associated with pancreatic carcinogenesis (author: Tomasz M. Karpiński).

The relative abundance of some bacteria may be decreased in patients with PDAC. *Neisseria elongata* [26,27] and *Streptococcus mitis* [27] were found to be less abundant in patients with PC when compared with healthy controls. By using a combination of both bacteria, Farrell et al. found that they were able to differentiate between chronic pancreatitis and PC with 85.7% sensitivity and 55.6% specificity. Farrell et al. likewise observed that levels of *Granulicatella adiacens* (increased in PC) and *S. mitis* (decreased in PC) differed significantly between patients with PC and those without cancer (including patients with chronic pancreatitis) [27]. By using both bacteria in combination as biomarkers, the authors were able to differentiate between PC patients and healthy controls with 96.4% sensitivity and 82.1% specificity. Lu et al. sequenced the tongue microbiota coating of patients with pancreatic head cancer (PHC), a PDAC found in the head of the pancreas, and healthy controls [28]. They found that significant differences exist between the two groups with respect to microbiota composition; specifically, PHC patients exhibited lower levels of *Haemophilus* and *Porphyromonas* and higher levels of *Leptotrichia* and *Fusobacterium* in their tongue microbiota coats. In contrast, Fan et al. found that oral carriage of *Fusobacteria* was associated with a decreased risk of developing PC (OR = 0.94) [23]. Carriage of the genus *Leptotrichia* was associated with an even greater decrease in the risk of developing PC (OR = 0.87). Bacterial associations related with pancreatic carcinogenesis are shown in Figure 1. Some of these bacteria and *Malassezia* fungus are presented as cultures in Figure 2.

The various aforementioned studies demonstrated that oral carriage of certain bacteria did not correlate with the development of PC. Fan et al. found that *Tannerella forsythia* and *Prevotella intermedia* were not associated with an increased risk of developing pancreatic cancer [23]. Additionally, Torres et al. found that the relative abundance of *S*. *mitis* and *G*. *adiacens* (both identified as potential biomarkers in other studies) did not differ between patients with PDAC and their healthy counterparts [26]. Furthermore, Lu et al. report they observed no difference in the relative abundance of Proteobacteria between patients with PHC and healthy controls [28]. Interestingly, the study likewise highlights that many of the differences in the relative abundance of various bacteria between the saliva of patients with PC and healthy controls do not extend to the tongue coat microbiome.

### 2.2. Gut

For some time, the association between gastric carriage of *Helicobacter pylori* and the development of PC has been known. A summary of meta-analyses conducted by Maisonneuve and Lowenfels calculated that *H*. *pylori* may be attributed to the development of 4% to 25% of all PC cases in Westernized countries [29]. Further highlighting the geographical differences between the association of *H*. *pylori* and PC is a study by Wang et al., which demonstrates that CagA+ subjects are more likely to develop the disease in Western, but not Eastern countries [45]. Furthermore, there are several studies and meta-analyses reporting positive associations between *H*. *pylori* infection and PC [30,31,32,33], as well as between CagA positivity and PC [34,35]. However, there are likewise several studies reporting no association between *H*. *pylori* infection and PC [35,36,37]. With the exception of these three studies, the majority of papers and meta-analyses examined during the present review found there to be an association between the presence of *H*. *pylori* and PC. Therefore, carriage of *H*. *pylori* may hold some value for potential screening protocols. Such screening measures would need to account for geography and the sensitivity and specificity of such tests would need to be determined in light of the limited associations.

Pushalkar et al., found that the relative abundance of *Proteobacteria*, *Synergistetes*, and *Euryachaeota* was significantly higher in the feces of patients with PDAC than in their healthy controls [38]. A small study conducted by Half et al., presented at a conference in Israel, highlights that the feces of patients with PC had higher levels of *Sutterela*, *Veillonela*, *Bacteroides*, *Odoribacter*, and *Akkermansia* than that of healthy controls [46]. Another study by Half et al. found that patients with PC had decreased levels of genera belonging to Firmicutes in their feces [47]. Studying overall gut microbial diversity, Ren et al. found that patients with PC had significantly reduced overall diversity [48]. Their study however failed to find any significant difference in microbial diversity between subtypes of PC (namely tumors located in the head of the pancreas vs. tumors located in the body and tail). Through the use of a murine model, Thomas et al. found that intestinal microbiota are important mediators of PC progression, with microbiota-depleted mice having decreased tumorigenicity [49]. Mendez et al. highlight that changes in the composition of fecal microbiota are evident early on in the course of tumor progression in a murine model of PDAC [50].

### 2.3. Pancreas

In addition to the potential association between gastric colonization by *H*. *pylori* and PC, several investigations have detected *H*. *pylori* in pancreatic tumors. A study of pancreatic tissue specimens by Nilsson et al. found that *Helicobacter* DNA was present in 48% of their tumor samples [39]. The authors also noted that only 5% of non-neoplastic tumors contained *Helicobacter* DNA when there was a nearby neuroendocrine or type 1 multiple endocrine neoplasia. There are several other intrapancreatic bacteria that have been associated with PC. According to Mitsuhashi et al., *Fusobacterium* species were found in 8.8% of their specimens, with *Fusobacterium*-positive patients having higher cancer-specific mortality rates [41]. On account of this, the authors suggest that screening for *F*. *nucleatum* in tumor samples may hold prognostic value, with patients positive for this species having a worse prognosis. More recently, Alkhaaran et al. demonstrated that patients with severe intraductal papillary mucinous neoplasm (IPMN) have higher levels of circulating IgG and salivary IgA reactive to *F*. *nucleatum* [42]. The positive correlation (r = 0.685, *p* < 0.0001) between *F*. *nucleatum* and Fap2 IgA antibodies is interesting on account of the fact that Fap2 is an important adhesin used by *F*. *nucleatum* to bind targets in the pancreatic TME [42,51,52]. A 2018 paper by del Castillo et al. likewise reported that a higher relative abundance of *Fusobacterium* spp. was observed in samples obtained from patients with PC [40]. Noncancer subjects had a higher relative abundance of *Lactobacillus* (µ = 0.06 vs. µ = 0.02 for PC patients, *p* < 0.0001).

An examination of human pancreatic tissue by Geller et al. revealed that the prevalence of bacterial DNA differed significantly between samples obtained from patients with PDAC and those of healthy controls (76% and 15%, respectively) [43]. Further 16S rDNA sequencing revealed that the most common bacterial class in the pancreatic tissue of PDAC patients is Gammaproteobacteria (specifically Enterobacteriaceae and Pseudomonadaceae). The above is supported by the work of Pushalkar et al., who noted that patients with PDAC have increased levels of gut Proteobacteria (specifically, *Pseudomonas* and *Elizabethkingia*) [38].

The role of the mycobiome as it relates to PC is only beginning to come under scrutiny. In the first study to examine the role of the mycobiome and its association with PC, Aykut et al. found that *Malassezia* is markedly increased in the pancreas of patients with PDAC [44]. The authors were able to further demonstrate that *Malassezia* promotes PDAC, with its ablation slowing tumor progression. *Candida*, *Saccharomyces*, and *Aspergillus* did not accelerate oncogenesis following pancreatic repopulation.

## 3. Oncogenic Mechanisms

### 3.1. Inflammatory Processes

It has long been known that inflammation plays a role in the development of cancer [53]. Certain oral pathogens such as *Porphyromonas*, *Prevotella*, and *Fusobacterium* may cause inflammation by contributing to the increase in inflammatory mediators such as IL-1β, IL-6, IL-17, IL-23, TNF-α, MMP-8, and MMP-9 [54].

Localized inflammatory processes may contribute to the development of neoplasia. Thus, the presence of bacteria in the pancreas may stimulate resident leukocytes to produce IL-1β upon contact with lipopolysaccharide (LPS) or other pathogen-associated molecular patterns (PAMPS). IL-1β is known to induce the production of proangiogenic factors associated with angiogenesis in the TME (e.g., VEGF, TNF) [55]. Furthermore, IL-1β is known to contribute to the metastasis [56,57] and aggressiveness [58,59] of neoplasms. Das et al. demonstrate that tumor-derived IL-1β is required for the establishment of the pancreatic TME, characterized by the activation of pancreatic stellate cells, and the induction of an immunosuppressive environment [60]. The authors report that the immunosuppressive milieu is specifically mediated by M2 macrophages, myeloid-derived suppressor cells, CD1d^hi^CD5^+^ regulatory B cells, and Th17 cells. This is in line with several other studies which have shown that IL-1β functions as an important modulator of TME immunosuppression [61,62]. In addition to the aforementioned leukocytes associated with immunosuppression, T lymphocytes likewise influence the progression of PC. In fact, Russano et al. found that through TCR sequencing it is possible to measure the heterogeneity of tumor-infiltrating T lymphocytes [63]. Moreover, this heterogeneity was found to reflect that of the mutational landscape within the tumor as well.

The proinflammatory cytokine IL-6 is likewise an important contributor to tumor growth and progression. Recent studies suggest that *H. pylori* may alter the expression levels of IL6 by means of miRNA regulation, particularly miR-195 and miR-488 [64,65]. IL-6 production may likewise be induced by contact between leukocytes and microorganisms such as bacteria or fungi [66,67]. By means of the JAK/STAT3 signaling cascade, IL-6 induces the expression of genes involved in cell proliferation and survival [68]. Moreover, the anti-apoptotic effects of IL-6 have been known for many years, and are thought to influence the development of various cancers [69,70]. Additionally, IL-6 has been implicated in the regulation of matrix metalloproteinases (MMPs) involved in the processes of tumor invasion and metastasis [71,72]. Interestingly, STAT3 induces the expression of IL6, thus creating a positive feedback loop, exacerbating tumorigenesis and metastasis [73].

**Figure 3:**
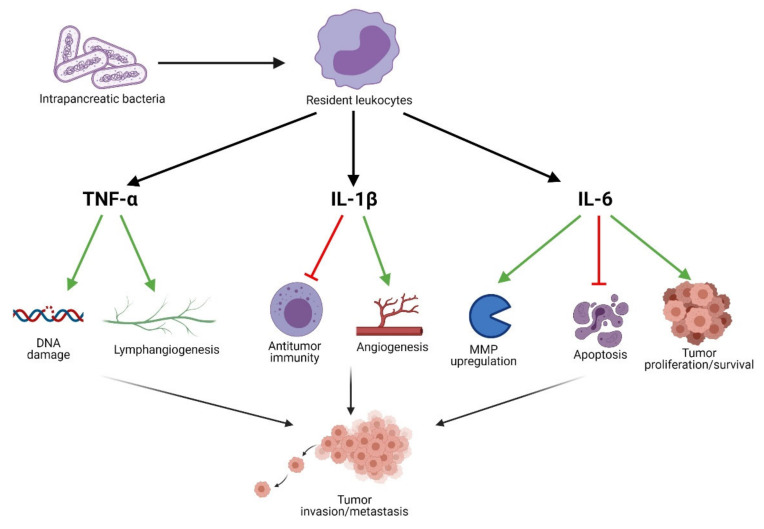
Inflammatory mediators contributing to pancreatic carcinogenesis.

TNF-α, another well-characterized inflammatory cytokine, is known to contribute to tumorigenesis when present at low concentrations [74]. The mechanism by which TNF-α is thought to induce tumorigenesis, relies on its induction of ROS and reactive nitrogen species (RNS), which in turn damage DNA [75]. Moreover, TNF-α has been found to play a role in metastasis by inducing lymphangiogenesis through VEGF-C and VEGF-D signaling in certain cancers [76,77]. In addition to the above, TNF-α likewise contributes to tumor invasiveness through NF-κB signaling [78]. The role of inflammatory mediators on pancreatic carcinogenesis is shown in Figure 3.

### 3.2. Translocation

Carcinogenesis may be enhanced by means of direct contact between bacteria and the tumor microenvironment (TME). Socransky and Haffajee calculated that individuals swallow approximately 10^11^ oral bacteria daily [79]. Furthermore, several studies have demonstrated that bacteria are able to translocate from the duodenum to the pancreas [50,51], offering a potential mechanism by which oral bacteria may come to colonize the pancreas. A review of pancreatic adenocarcinomas in the USA over a four-decade period revealed that most cases of the neoplasm were present in the head of the pancreas [80]. Thomas and Jobin suggest that a potential explanation for this may lie in the close association of the pancreatic head with the duodenum, thus being the first site exposed to translocating bacteria [81]. In addition to retrograde translocation through the GI tract, Mitsuhashi et al. outline a series of studies which suggest that some bacteria (namely *F*. *nucleatum*) may reach the pancreas via circulation [46].

For some years, *F*. *nucleatum* has been known to contribute to the modulation of the TME in various gastrointestinal neoplastic diseases. *Fusobacterium* adhesin A (FadA) enables *F*. *nucleatum* to bind host cells via interactions with cadherins [82]. Kostic et al. found that tumor-infiltrating myeloid cells are selectively recruited by *F*. *nucleatum* thus promoting tumor development by generating a pro-inflammatory microenvironment [83]. Specifically, this may include the generation of reactive oxygen species (ROS). These free radicals are directly responsible for DNA damage implicated in a number of characteristic features of cancer (e.g., cell growth, proliferation, genomic instability) [84]. In addition to DNA damage, ROS modulate the activity of tumor-infiltrating leukocytes (TILs), creating an immunosuppressive environment favorable for tumor progression [85]. Moreover, mast cells affect not only the development and progression of PDAC, but as noted by Porcelli et al., actively contribute to resistance against chemotherapeutics, notably gemcitabine/nabpaclitaxel [86]. Their study found that the mast cell mediated effects occur as a result of activation of T*βRI signaling, promoting tumor cell regrowth.* This suggests that tumor-infiltrating myeloid cells contribute to the development, progression, and resistance to treatment observed in patients with PDAC. Additionally, the virulence factor familial adenomatous polyposis 2 (Fap2) interacts with the receptor T cell immunoreceptor with Ig and ITIM domains (TIGIT) on NK cells and lymphocytes, suppressing their cytotoxic functions [87]. Chen et al. report that *F*. *nucleatum* upregulated the expression of long non-coding RNA Keratin7-antisense (*KRT7*-*AS*) via the NF-κB pathway both in vitro and in an in vivo murine model of colorectal cancer [88]. Such long non-coding RNAs have been found to play an essential role in metastasis.

**Figure 4:**
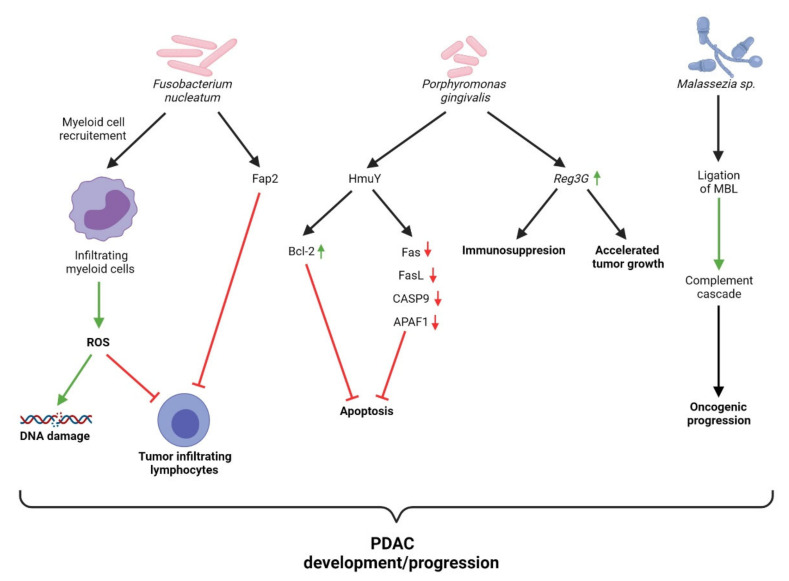
Overview of mechanisms of bacterial-mediated carcinogenesis of pancreas.

The association between *P*. *gingivalis* and PC is well established, with a number of molecular mechanisms behind this association garnering increasingly more supporting evidence. *P*. *gingivalis* has been associated with a number of systemic diseases, with much of its pathogenic potential being attributed to the suppression of adaptive immune responses [89]. An in vitro model utilized by Gnanasekaran et al. demonstrates that *P*. *gingivalis* survives within pancreatic cancer cells and enhances their proliferation [90]. Additionally, Carvalho-Filho et al. demonstrated that PBMCs from patients with periodontitis more greatly downregulated genes associated with apoptosis than their healthy counterparts, when exposed to the *P*. *gingivalis* HmuY protein [91]. This study is in line with the author’s previous work which highlighted that HmuY increased the expression of Bcl-2, an anti-apoptotic protein, in CD3^+^ T cells [92]. Moreover, in a murine model, Hiraki et al. demonstrated that intraperitoneal administration of *P*. *gingivalis* LPS upregulated the expression of regenerating islet-derived 3G (*Reg3G*) in pancreatic tissue [93]. The overexpression of *Reg3G* has been found to accelerate tumor growth and induce an immunosuppressive microenvironment [94]. Mechanisms of bacterial-mediated carcinogenesis of the pancreas are presented in Figure 4.

The contribution of fungi to the development and progression of PC has only recently begun to be investigated. The seminal paper by Aykut et al. highlights several key important findings relating to *Malassezia* and PC [52]. The study reports that the relative abundance of *Malassezia* is markedly increased in the pancreas compared with the gut in both patients and mice with PC. Fungal ablation in the study’s murine model was found to decrease oncogenic progression, while repopulation with *Malassezia globosa* accelerated PDAC progression. Mannose-binding lectin (MBL) is responsible for the initiation of the lectin-pathway of complement activation [95]. Aykut et al. were able to demonstrate that the expression of *MBL* was associated with reduced survival in human PDAC patients, while knockdown of the pathway proved to mitigate tumor growth in their murine model. These results demonstrate that the MBL-mediated complement activation pathway is involved in pancreatic oncogenesis. With regards to other fungal species, Kaźmierczak-Siedlecka et al. outline several oncogenic mechanisms which have been identified; however, the results are confined to non-pancreatic cancers [96]. The scarcity of research pertaining to the role of fungi in relation to PC highlights the urgent need for more research.

## 4. Conclusions

There are a number of microorganisms which are associated with the development and progression of PC. Those with the most literature supporting such associations are *Porphyromonas gingivalis*, *Fusobacterium nucleatum*, and *Helicobacter pylori*. The mechanisms involved in this bacterial-mediated carcinogenesis include inflammatory mediators, immune cells, reactive oxygen species, and modulation of genes associated with apoptosis and growth/proliferation. The role of fungi in the development and progression of PC is only beginning to come under investigation and as such may reveal as yet unknown associations and mechanisms. The work of Aykut et al. with *Malassezia* highlights the important role fungi may play in carcinogenesis, calling attention to the lack of research connecting the mycobiome to cancer. The study of such microbial associations with PC may lead to the discovery of highly sensitive and specific changes which can serve as biomarkers for the purpose of screening larger proportions of the population. This in turn may help to alleviate the problems presented by low early detection rates in the general population. Furthermore, understanding the microbial basis for PC development and progression may prove to be beneficial for prophylactic measures and the establishment of new treatment guidelines. This may involve targeting specific microorganisms to abolish carrier status or attenuating their numbers in at-risk individuals.

Studies such as that by Guo et al., which found that the ratio of *F*. *nucleatum* to *Bifidobacterium* could detect colorectal cancer with 84.6% sensitivity and 92.3% specificity, show promise for the use of microorganisms as biomarkers in cancer screening [97]. Unfortunately, there is not yet enough information available to utilize microorganisms as biomarkers for the purpose of screening for PC. Even microbial populations exhibiting strong associations with cancer (e.g., *H*. *pylori*, *P*. *gingivalis*) fail to provide actionable diagnostic information for PC when examined in isolation. With the exception of the aforementioned 2012 paper by Farrell et al., research for the present review failed to find any subsequent work considering multiple microorganisms in tandem for diagnostic purposes relating to PC. Future work relating to biomarkers should take into account several bacterial and fungal species together to assess if they hold increased prognostic/diagnostic value when examined in tandem.

## Figures and Tables

**Table 1:** Microbial associations with pancreatic cancer.

Microbial Population	Microbial Localization	Main Finding ^‡^	Methods	Sample Size *	Reference
*Porphyromonas gingivalis*	Oral	Increased association with PC OR = 1.60 [CI = 1.15–2.22, P = NR] OR = 2.14 [CI = 1.05–4.36, P = NR]	16S rRNA	P = 361 H = 371	[23]
			Immunoassay	P = 405 H = 416	[24]
*Aggregatibacter actinomycetemcomitans*	Oral	Increased association with PC OR = 2.20 [CI = 1.16–4.18, P = NR]	16S rRNA	P = 361 H = 371	[23]
*Alloprevotella* sp.	Oral	Increased association with PC OR = 1.20 [CI = 1.01–1.43, P = NR]	16S rRNA	P = 361 H = 371	[23]
*Streptococcus* sp.	Oral	Increased association with PC OR = 5.344 [CI = 1.282–22.282, *p* = 0.021]	16S rRNA	P = 41 H = 69	[25]
*Veillonella* sp.	Oral	Decreased association with PDAC OR = 0.187 [CI = 0.055–0.631, *p* = 0.007]	16S rRNA	P = 41 H = 69	[25]
*Neisseria* sp.	Oral	Decreased association with PDAC OR = 0.309 [CI = 0.100–0.952, *p* = 0.041] NS [*p* = 0.07] Significantly lower in PC [*p* < 0.05]	16S rRNA	P = 41 H = 69	[25]
			16S rRNA	P = 8 O = 78 H = 22	[26]
			16S rRNA	P = 10 H = 10	[27]
*Streptococcus mitis*	Oral	Decreased association with PDAC Lower in PC [P < 0.05]	16S rRNA	P = 10 H = 10	[27]
*Gemella adiacens*	Oral	Increased association with PDAC Higher in PC [*p* < 0.05]	16S rRNA	P = 10 H = 10	[27]
*Haemophilus* sp.	Oral	Decreased population in PHC Lower in PHC [*p* < 0.001]	16S rRNA	P = 30 H = 25	[28]
*Porphyromonas* sp.	Oral	Decreased population in PHC Lower in PHC [*p* < 0.05]	16S rRNA	P = 30 H = 25	[28]
*Fusobacterium* sp.	Oral	Increased population in PHC Higher in PHC [*p* < 0.05] Decreased association with PC OR = 0.94 [CI = 0.89–0.99, *p* = 0.014]	16S rRNA	P = 30 H = 25	[28]
			16S rRNA	P = 361 H = 371	[23]
*Leptotrichia* sp.	Oral	Increased association with PDAC OR = 6.886 [CI = 1.423–33.357, *p* = 0.0016]	16S rRNA	P = 41 H = 69	[25]
		Increased population in PHC Higher in PHC [*p* < 0.01] Decreased association with PC OR = 0.87 [CI = 0.79–0.95, *P* = 0.0029]	16S rRNA	P = 30 H = 25	[28]
			16S rRNA	P = 361 H = 371	[23]
*Helicobacter pylori*	Gut	Increased association with PC Population attributable fraction = 4–25% [P = NR]	Averaging results of 3 meta-analyses	Literature review	[29]
		Increased association with PC Seropositivity in PC patients = 65% [P = NR]	Immunoassay for IgG	P = 92 O = 65 H = 20	[30]
		Increased association with PC OR = 1.45 [CI = 1.09–1.92, *p* = 0.032]	Meta-analysis of 8 studies	P = 1003 H = 1754	[31]
		Increased association with PC Association between seropositivity and PC OR = 1.38 [CI = 1.08–1.74, *p* = 0.009]	Meta-analysis of 6 studies	P = 2335 H = unknown	[32]
		Increased association with PC OR = 1.47 [CI = 1.22–1.77, P = NS]	Meta-analysis of 9 studies	P = 1083 H = 1950	[33]
		Increased association between CagA positivity and PC Higher in PC [38.88 vs. 21.53%, *p* < 0.05]	Immunoassay for CagA	P = 56 H = 60	[34]
		No association between *H*. *pylori* and PC OR = 0.99 [CI = 0.65–1.50, P = NS] OR = 1.00 [CI = 0.74–1.35, P = NS] OR = 1.09 [CI = 0.81–1.47, P = NS]	Meta-analysis of 5 studies	P = 1446 H = 2235	[35]
			Immunoassay for *H*. *pylori*	P = 580 H = 626	[36]
			Meta-analysis of 6 studies	P = 1894 H = 2683	[37]
		Increased association between CagA and PC OR = 1.42 [CI = 0.79–2.57, *P* = 0.001]	Meta-analysis of 5 studies	P = 1083 H = 1950	[33]
		Geographical differences affect association between *H*. *pylori* and PC development Western countries: OR = 1.14 [CI = 0.89–1.40, P = NS] Eastern countries: OR = 0.62 [CI = 0.49–0.76, P = NS]	Meta-analysis of 9 studies	P = 2049 H = 2861	[30]
Proteobacteria	Gut	Elevated relative abundance in PC [*p* < 0.05]	16S rRNA	P = 32 H = 31	[38]
Synergistetes	Gut	Elevated relative abundance in PC [*p* < 0.001]	16S rRNA	P = 32 H = 31	[38]
Euryarchaeota	Gut	Elevated relative abundance in PC [*p* < 0.001]	16S rRNA	P = 32 H = 31	[38]
*Elizabethkingia* sp.	Gut	Elevated relative abundance in PC [P = NR]	16S rRNA	P = 32 H = 31	[38]
*H*. *pylori*	Pancreas	*H*. *pylori* found in 48% of pancreatic tumors [P = NR]	16S rDNA	P = 40 O = 10 H = 7	[39]
*Fusobacterium* sp.	Pancreas	Increased association with PC [*p* < 0.0001]	16S rRNA	P = 50 H = 34	[40]
		Fusobacterium present in pancreatic tumor is associated with higher mortality [17.2 vs. 32.5 months median survival time, *p* = 0.023]	PCR (seq not provided)	P = 283 H = N/A	[41]
		Increased associated with severe IPMN [*p* = 0.007]	Immunoassay for salivary IgA	P = 65 H = 8	[42]
*Lactobacillus* sp.	Pancreas	Decreased association with PC [*p* < 0.0001]	16S rRNA	P = 50 H = 34	[40]
Enterobacteriaceae	Pancreas	Increased association with PC 76% of PDAC samples vs. 15% of control samples [*p* < 0.005]	16S rRNA	P = 113 H = 20	[43]
Pseudomonadaceae	Pancreas	Increased association with PC 76% of PDAC samples vs. 15% of control samples [*p* < 0.005] [P = NR]	16S rRNA	P = 113 H = 20	[43]
			16S rRNA	P = 32 H = 31	[38]
Malassezia sp.^†^	Pancreas	Elevated relative abundance in PC [P = NR]	18S rRNA	P = 13 H = 5	[44]

* P, pancreatic cancer (includes PHC, PDAC, and general PC); H, healthy control; O, other pancreatic disease. ^‡^ Statistical values are listed in the order of references in a given row; NR, not reported; NS, non-significant; odds ratios are presented with 95% confidence intervals. ^†^ Malassezia is a fungal genus, all other microbial populations in the table are bacterial.
